# The views, opinions and decision-making of UK-based paramedics on the use of pre-hospital 12-lead electrocardiograms in acute stroke patients: a qualitative interview study

**DOI:** 10.29045/14784726.2023.12.8.3.1

**Published:** 2023-12-01

**Authors:** Scott Munro, Debbie Cooke, Janet Holah, Tom Quinn

**Affiliations:** South East Coast Ambulance Service NHS Foundation Trust; University of Surrey ORCID iD: https://orcid.org/0000-0002-0228-4102; University of Surrey ORCID iD: https://orcid.org/0000-0003-1944-7905; South East Coast Ambulance Service NHS Foundation Trust; Kingston University ORCID iD: https://orcid.org/0000-0002-5116-0034

**Keywords:** decision-making, emergency medical services, stroke

## Abstract

**Introduction:**

A qualitative exploration into the views, opinions and decision-making of paramedics involved in undertaking pre-hospital 12-lead electrocardiograms (PHECGs) for stroke patients was undertaken, in order to gain a deeper understanding of the clinical and occupational context that the paramedics work within, the acceptability of the paramedics in using PHECGs for stroke patients and the consequences and influences of their decision-making.

**Methods:**

Data were collected via semi-structured interviews and analysed using the framework method, with the underpinning theoretical framework of cognitive continuum theory. A purposive sample of 14 paramedics was recruited and interviewed.

**Results:**

Five themes were generated from the analysis of the interviews: (1) ‘time is brain’: minimising delays and rapid transport to definitive care; (2) barriers and facilitators to undertaking PHECGs for stroke patients; (3) recognising and gaining cues; (4) maintaining patient dignity, self-protection and fully informed consent; and (5) education, experience and engagement with evidence.

**Conclusion:**

The study showed mixed views on the usefulness of PHECGs, but all participants agreed that PHECGs should not cause additional delays. Paramedic decision-making on recording PHECGs relies on intuitive and quasi-rational cognitive modes, and requires a number of clinical, logistical and ethical considerations. The findings suggest careful consideration is needed of the benefits and potential drawbacks of incorporating PHECGs into pre-hospital stroke care.

## Introduction and background

Emergency medical services (EMS) play a crucial role in the pre-hospital recognition, management and transportation of acute stroke patients (Cash et al., 2022). Despite improvements in reducing delays from arrival at hospital to treatment, pre-hospital delays remain a significant challenge. In the United Kingdom, stroke systems and EMS have implemented several measures in attempts to reduce pre-hospital delays and improve patient outcome, such as the use of pre-alerts (Sheppard et al., 2015). However, time-consuming procedures such as pre-hospital 12-lead electrocardiograms (PHECGs) contribute to on-scene time. Studies have demonstrated that stroke patients who receive a PHECG spend an average of 4–7 minutes longer on scene with EMS, with no value on patient outcome gained (Drenck et al., 2019; McClelland et al., 2023; Munro et al., 2022).

Current guidelines for UK EMS recommend avoiding interventions that do not add value to the management of stroke patients, such as PHECG, unless clearly indicated (Association of Ambulance Chief Executives, 2021). However, despite the lack of evidence supporting the routine use of PHECGs in stroke patients, they continue to be used in practice. One study reported 48% (558/1161) of stroke patients receiving a PHECG (Munro et al., 2022), while 68% of respondents of a survey of UK paramedics’ views of stroke training and current practice stated that they routinely undertake PHECGs on stroke patients (McClelland et al., 2017). Therefore, understanding the perspectives and decision-making processes of stakeholders involved in PHECGs for stroke patients is crucial in ensuring optimal pre-hospital care for this patient group.

This study aimed to explore the views, opinions, attitudes and decision-making of paramedics involved in undertaking PHECGs for stroke patients, in order to gain a deeper understanding of the clinical and occupational context that the paramedics work within, the acceptability of using PHECGs for stroke patients and the guidelines that recommend their use, as well as the cognitive strategies and influences of their decision-making. The study was conducted as part of a concurrent mixed-methods study investigating the use and impact of PHECGs in acute stroke patients.

The research questions addressed by this study were:

- What are the views, attitudes and perceived value of PHECGs in acute stroke patients, from the paramedics involved in their care?
- How do paramedics make decisions regarding PHECGs for acute stroke patients, and what influences these decisions and cognitive strategies?

## Methods

This study is reported following the consolidated criteria for reporting qualitative research (COREQ) guidelines (Tong et al., 2007).

### Theoretical underpinning

Cognitive continuum theory (CCT) was used as the underpinning theoretical framework to explore the decision-making employed surrounding the use of PHECGs (Hamm, 1988; Hammond, 2000; Standing, 2008). CCT states that cognition is a continuum that ranges from intuitive to analytical, with various task factors influencing the extent to which one relies on intuition or analysis (Box 1) (Parker-Tomlin et al., 2017; Standing, 2008). Task structures vary from ill structured to well structured, with ill-structured tasks inducing more intuitive cognition, and conversely, well-structured tasks inducing analytic cognition. Quasi-rationality relates to a mode of cognition that has elements of both intuition and analysis (Cader et al., 2005).

**Figure:**
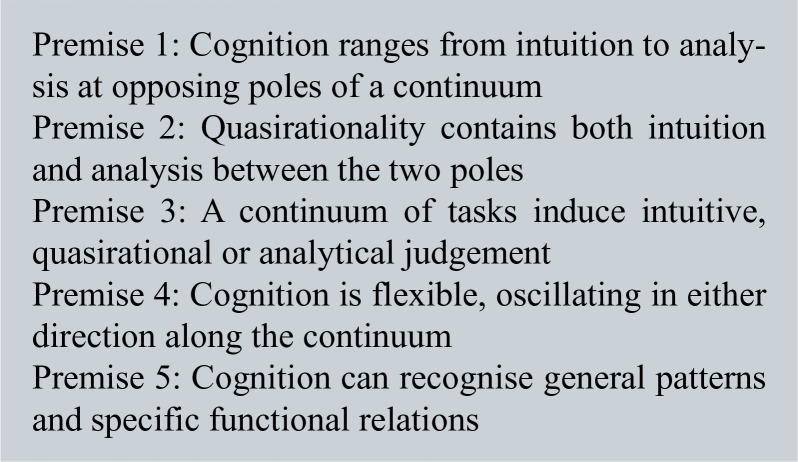
Box 1. The five premises of cognitive continuum theory.

Source: Standing, 2008.

### Data collection and participants

Data were collected through semi-structured interviews conducted by SM, as part of SM’s PhD thesis, while working as a paramedic for the participating EMS. Participants were recruited using a mix of purposive, opportunistic and snowball sampling. Interviews were conducted face to face at a location chosen by the participant, and were audio-recorded and transcribed verbatim by SM. No one else was present during the interviews besides the participant and SM.

An interview topic guide was developed by SM, DC, JH and TQ, following an iterative process that involved modifying the questions depending on the analysis of the first few interviews. No repeat interviews were conducted, and data collection was considered complete when informational redundancy was achieved. Rather than a discrete event, this was an incremental approach where, although there was the potential for more new data to be generated, it would not have necessarily contributed or added to the overall story of the study (Saunders et al., 2018).

The framework method (Ritchie & Lewis, 2003) was used to produce an exploratory analysis for the data collected (Box 2). The initial three interviews were coded independently by SM and DC, using inductive coding. SM, DC and TQ then developed an analytic framework, and SM applied it to the remaining interviews.

**Figure:**
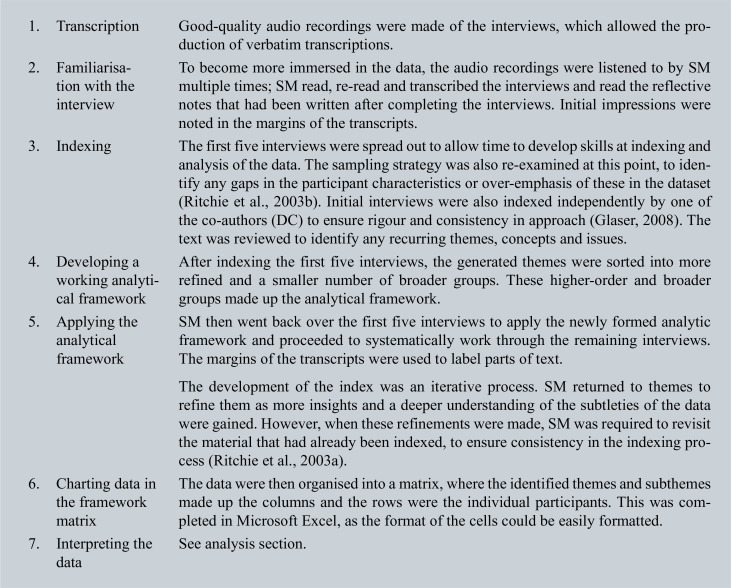
Box 2. Framework method.

### Analysis

A total of 14 paramedics were recruited from one EMS system in the south of England (see Table 1). Five themes were generated from the interviews (see Box 3).

**Table 1.** Characteristics of paramedic participants.

ID	Age	Sex	Ethnicity	Years of experience	Education/training background
Paramedic 1	20–24	Male	White British	0–4	Higher education
Paramedic 2	25–29	Female	White British	5–9	Higher education
Paramedic 3	30–34	Female	White British	0–4	Higher education
Paramedic 4	25–29	Male	White British	0–4	Higher education
Paramedic 5	30–34	Female	White British	0–4	Higher education
Paramedic 6	45–49	Female	White British	10–14	IHCD
Paramedic 7	20–24	Male	White British	0–4	Higher education
Paramedic 8	40–44	Male	White British	15–19	IHCD and higher education
Paramedic 9	45–49	Male	White British	15–19	IHCD
Paramedic 10	35–39	Male	White British	15–19	IHCD
Paramedic 11	35–39	Male	White British	15–19	IHCD
Paramedic 12	55–59	Female	White British	10–14	IHCD
Paramedic 13	35–39	Male	White British	10–14	IHCD
Paramedic 14	25–29	Male	White British	5–9	Higher education

IHCD: Institute of Health and Care Development.

**Figure:**
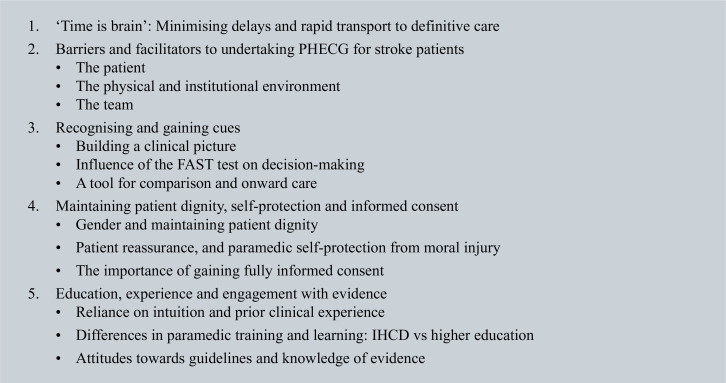
Box 3. Generated themes.

### Theme 1: ‘time is brain’ – minimising delays and rapid transport to definitive care

All participants recognised the time-critical nature of stroke and prioritised minimising delays to improve patient outcomes. However, opinions differed regarding whether the PHECG was a necessary assessment tool or a potential cause of delay. Participants recognised the importance of minimising pre-hospital time while considering the impact of any assessments or interventions on patient management.

*as soon as that’s flagged up by a relative and as soon as that patient is FAST positive and you deem it to be a stroke, then your job is to get to hospital as quickly as possible to save as much brain tissue as possible. Personally, that’s my view on what the ambulance service’s role is . . . Time is brain!* (Paramedic 1)

### Theme 2: barriers and facilitators to undertaking pre-hospital 12-lead electrocardiograms for stroke patients

The participants discussed that there were certain barriers, tensions and facilitators that influenced their decision-making around whether undertaking a PHECG was going to take a long time or whether it could be done quickly.

#### The patient

There was a multitude of challenges described that could affect the time it would take to undertake a PHECG, due to the patients’ characteristics, such as layers of clothing, body weight, ability to obey commands and severity and ambiguity of symptoms. The participants discussed having to take these into consideration when making decisions about whether to record a PHECG or not.

#### The physical and institutional environment

The physical environment in which to record the PHECG was described as an important consideration, and opposing views among the participants surrounding the location of the incident in relation to the hospital were discussed. One participant suggested that if the incident was a long distance from hospital, they would not want to add on further delay by undertaking a PHECG. Conversely, another participant stated that if the location was close to a hospital, it may be more beneficial to quickly transport the patient there, rather than take the extra time to undertake a PHECG.

*If you’re a long way away from hospital, you don’t want to spend that extra 3 minutes doing that ECG at the beginning.* (Paramedic 12)

*If you are 200 yards from the hospital, I suppose you have to weigh it up at that time. Maybe it would influence my decision not to do one . . . they’re 200 yards from the hospital, then that is the best place if you are querying a stroke. For the sake of an extra 10 minutes on the scene, 45 seconds to hospital, plus unloading, yes that might sway my decision not to do one.* (Paramedic 10)

Tensions within the paramedic’s working and institutional environment were also discussed as having an influence on decision-making. One of the participants recalled experiences of being disciplined for taking too long on scene and told not to record PHECG for stroke patients.

*There are a couple of times working . . . because our scene time, they had been delayed . . . that was the one where we did do an ECG. The delayed scene time had been picked up via clinical audit from the [patient clinical record] and we did get questioned about it. And we got questioned in, what I feel, was a fairly negative way, and we had to justify.* (Paramedic 3)

#### The team

The configuration of EMS crews and authority gradients was found to influence decision-making. Paramedics working alone on a single response vehicle may opt to perform a PHECG while waiting for a double-crewed ambulance to transport the patient, without causing further delay. Hierarchy, seniority and clinical rank were also identified as factors affecting collaborative decision-making. Paramedics were less likely to engage in such discussions with colleagues of higher or lower clinical grades.

*If a senior clinician said, ‘oh, I always do one’, I would probably go along with them. If they’re not senior . . . we’ll have a quick discussion and then we’d make our mind up as to what we’re gonna do. If they’re junior I’d probably say, ‘just stick with what I’m gonna do’. If we’re the same level, I would have a discussion and say, ‘you know I wouldn’t do it because of this, do you agree?’.* (Paramedic 7)

### Theme 3: recognising and gaining cues

#### Building a clinical picture

Paramedics’ decision-making on whether to record PHECGs was discussed to be influenced around both recognising present pertinent cues and realising that more cues were necessary to help build a ‘clinical picture’. The analogy of the PHECG being a ‘part of the puzzle’ to help complete this picture was used.

*You’re trying to build up the big picture about what’s going on with the patient . . . Not going too in depth, but I would say that I would use the ECG as a diagnostic tool to try and figure out what’s going on.* (Paramedic 13)

#### Influence of the face, arm, speech test on decision-making

The participants discussed how certain clinical presentations of patients provide cues of differing weight that influence their decision about whether it was important to record a PHECG or not. The findings from a face, arm, speech test (FAST) (Harbison et al., 2003) were discussed as having an influential role in the decision-making of whether to record a PHECG. A FAST positive finding was considered a strong determinant to not record a PHECG for some participants, as this led them directly to a differential diagnosis of stroke. However, if the patient was FAST negative, this may influence the paramedics’ decisions in favour of performing a PHECG, as it may then be considered as a valuable tool for gaining more cues to help build a clinical picture of what was happening to the patient.

*because in the ones where it’s FAST positive and it’s obvious . . . I know what the outcome for that patient is gonna be. We’re going to be going to hospital. The more subtle ones . . . then I’m more likely to do an ECG in those patients . . . I would more likely to do it in the ones who have more subtle symptoms, rather than the obvious.* (Paramedic 3)

#### A tool for comparison and onward care

The participants discussed how the information gathered from the PHECG may not be directly useful to the paramedic crew but may be of use later in the patient’s ‘journey’. The PHECG may be useful to compare against electrocardiograms (ECGs) recorded in hospital to see if any changes have occurred. Collecting data in the pre-hospital phase could enable the documentation of transient clinical signs or symptoms that might not be present upon the patient’s arrival at the hospital. It may be the only opportunity for an abnormality to be recorded, and this could have implications on the patient’s continuing care.

*It [PHECG] helps the patient’s journey. I think paramedics are very focused on, I spend one hour with this patient and then that’s their whole interaction, whereas the patient has the rest of their life, essentially, if it’s a stroke, with that event, so . . . you’ve got to make sure you can get some information early on, which may help them in the future, if you’ve got time.* (Paramedic 5)

### Theme 4: maintaining patient dignity, self-protection and fully informed consent

#### Maintaining patient dignity and gender

Some of the paramedic participants spoke of being conscious of maintaining patient dignity when undertaking PHECGs. The sex of the EMS crew and the patient was discussed as a consideration when making decisions around maintaining patient dignity and privacy when undertaking PHECG in stroke patients. Maintaining the dignity of women was a more dominant theme discussed by the male interviewees. The gender mix of the EMS crew was also discussed as an influence in the decision of the paramedics as to whether to undertake a PHECG.

Paramedic 12 (female participant) noted that female paramedics are perceived to have greater social acceptability when applying PHECGs to female patients, but in critical situations, patient concern is not focused on the sex of the paramedic.

*I think if you have a crew that is male/female, the males usually defer to the female to put the dots on, because it seems to be more polite, more acceptable and probably nicer for the female, because it’s not a stranger. I think it’s prudent to use your female crew mate to do that, but most women who are really, really unwell would not really care whether it was a man or a woman doing it, as long as they get consent.* (Paramedic 12)

#### Patient reassurance and paramedic self-protection from moral injury

Some of the participants suggested that the act of undertaking a PHECG gives the impression that the paramedic is actively engaged in patient care, which could reassure the patient and improve their overall experience, even if the paramedic believed it did not contribute to their assessment or management of the patient. This was to create a facade of activity. Additionally, some participants felt that doing a PHECG was a form of coping mechanism to overcome the perceived inability to actively help the patient during their pre-hospital care. Doing the PHECG gave them a sense of purpose.

*I want to feel like I’m doing something. So, it’s a lot of the time, with a stroke, I’m not able to administer definitive care, so just sitting there and watching somebody suffer, that’s not a nice experience for me, so you know, that personal awareness as well, from wanting to do something . . .* (Paramedic 14)

#### The importance of gaining fully informed consent

Due to the physical nature of placing the ECG dots on patients’ chests, gaining informed consent was considered important by the interviewees. However, due to the potentially debilitating nature of the disease, the participants described how patients may not have the ability to be able to give proper informed consent. The paramedic participants felt it was sometimes necessary to make decisions in the ‘best interest’ of the patient, for those who were not able to give informed consent.

Paramedics also discussed involving the patient’s family members or partners in decision-making if the patient is unable to give informed consent. They discussed adopting a collaborative approach to decision-making around undertaking a PHECG.

### Theme 5: education, experience and engagement with evidence

#### Reliance on intuition and prior clinical experience

Some participants spoke of using intuition and drawing from previous experiences as a strong influence in deciding how important a PHECG for stroke patients was. They were not able to provide a comprehensive answer to why they would decide which patients they would record a PHECG on, other than tacit and intuitive knowledge, based on previous experience.

*You just have this feeling that something’s going on . . . Intuition with the experience you’ve had.* (Paramedic 12)

#### Differences in paramedic training and learning: Institute of Health and Care Development versus higher education

A frequently mentioned topic was the conflicting beliefs as to whether there were differences in the practice of recording PHECGs between paramedics who trained through the Institute of Health and Care Development (IHCD) and those who trained as part of a higher education institution. While some believed that differences in practice existed, others argued that this was too broad a generalisation. One paramedic suggested that understanding the current evidence was a stronger factor in influencing how paramedics approached their practice.

*I think those that are aware of literature that exists around it are going to be more likely to undertake such a test [PHECG], but to differentiate between whether that’s more likely to be an IHCD or the degree training is quite harsh . . . But I think that would be your influence is if they are well read around the literature around the subject, rather than necessarily the training that they did.* (Paramedic 14)

#### Attitudes towards guidelines and knowledge of evidence

There were conflicting responses regarding the ‘Joint Royal Colleges Liaison Committee (JRCALC) clinical guidelines’ (Association of Ambulance Chief Executives, 2021), which provides guidance for NHS paramedics. Some participants felt that these guidelines should be strictly adhered to, with one of the participants referring to the guidelines as ‘our Bible’, while others emphasised the fact that they are ‘guidelines’, there to guide rather than dictate.

*I think it’s important that we should follow JRCALC. I follow the JRCALC, it’s our Bible. So, without that, I don’t know.* (Paramedic 11)

Even though evidence and research were considered important in informing their practice, many of the participants admitted that they had little knowledge of the evidence base surrounding PHECG for stroke patients. Others felt that the perceived lack of evidence to guide pre-hospital care was a limiting factor in being able to provide evidence-based care.

## Discussion

In this qualitative study, paramedics involved in the care of acute stroke patients were interviewed about their views, opinions, practices and decision-making regarding pre-hospital ECGs for stroke patients. While there were mixed views about the importance and usefulness of PHECGs for pre-hospital management of stroke patients, all participants agreed that these tests should not contribute to any delays in reaching the hospital. The study highlights the need for a balanced approach to patient care that takes into account the time-critical nature of stroke, and the impact of assessments and interventions on patient management. This is in line with Campeau’s (2009) ‘space-control theory’, which describes the element of ‘temporality at the scene’. This states that paramedics’ actions at the scene of an incident are dominated by the passage of time. Paramedics have to organise their activities in consideration of the clinical urgency of the patient, the uncertainty of diagnosis and prognosis, management oversite and scene circumstances. Overall, this study emphasises the importance of timely and efficient pre-hospital management of stroke patients, and highlights the need for a comprehensive and patient-centred approach to care that prioritises both the clinical urgency of the patient and the impact of assessments and interventions on patient outcomes.

The strategies used by paramedics in making judgements and clinical decisions regarding PHECGs for stroke patients have implications for the use of CCT as a theoretical framework to understand their decision-making processes. The paramedic participants reported using experience and intuition when there was a lack of time, resources and evidence to use more analytic cognitive strategies. While intuitive thinking has been criticised for its reliance on heuristics and potential for bias (Tversky & Kahneman, 1974), CCT suggests that it can be useful when matched correctly with the structure of the task at hand (Standing, 2010). The paramedic participants described managing stroke patients as what would be considered a low-structured task, working in dynamic and uncertain environments, where there may be limited information available about the patient’s condition, and where there is a need to make rapid and complex decisions in a time-sensitive context and a lack of evidence that can be easily applied to the situation. Instead, paramedics described having to rely on their experience, intuition and judgement to make the best decisions possible based on the information available to them. The study demonstrates that paramedics accurately align intuitive and quasi-rational cognitive strategies with the low-structured task of managing acute stroke patients.

The social/institutional context was also described to have had an impact on decision-making. The participants discussed issues of hierarchy and its influence on the decision-making process of whether to record a PHECG. EMS have been previously described as hierarchically structured organisations with informal power relations (Post et al., 1997). Wankhade (2012) has stated how paramedics and clinical staff within EMS systems must deal with the negative effects of hierarchy when dealing with unpredictable events, which can have implications for patient safety. This finding highlights the importance of further investigating power differentials within EMS teams to ensure effective and safe decision-making.

The participants discussed the potential usefulness for PHECGs to act as a baseline for comparison to ECGs recorded later in a patient’s hospital stay. Studies have found that PHECGs may detect paroxysmal atrial fibrillation (AF) that may be missed during hospital monitoring (Bobinger et al., 2017). Lykhage et al. (2020) reported in their study of continuous PHECG monitoring through only limb lead II, that 1 in 16 AF cases would have been missed. The ability to capture AF through means other than a full 12-lead ECG was not discussed by the participants, but may be a solution to mitigate some of the barriers discussed during the interviews and provide useful clinical data for the early diagnosis of AF, initiation of treatment and improved prognosis following stroke (Chou et al., 2018; Lykhage et al., 2020).

Paramedics using PHECG for stroke patients may face ethical concerns that can result in conflicting values and norms in treating patients. In this study, male paramedics were found to be more concerned about gender-related issues than female paramedics. However, a survey of women’s attitudes towards ECGs revealed that 94% of participants were willing to consent to PHECG regardless of the sex of the EMS crew (Wallen et al., 2013). These findings suggest that male paramedics may face more issues related to gender and patient dignity, while patients prioritise receiving care quickly over gender concerns related to PHECG recording. Prior studies have shown that potential gender bias affects the administration and timing of emergency care (Blewer et al., 2018; Lewis et al., 2019), particularly for younger female stroke patients who are less likely to receive a PHECG (Munro et al., 2022).

Paramedics in this study acknowledge the importance of evidence-based care, but feel limited by a lack of individual knowledge and perceived gaps in the evidence base, which forces them to rely on intuitive decision-making. A survey of Australian paramedics found that 90% believe that evidence-based care can have a positive impact on patient care, and 98% would change their clinical practice based on research from the pre-hospital environment (Simpson et al., 2012). Further, qualitative interviews conducted by Ankolekar et al. (2014) with paramedics participating in a pilot study of the rapid intervention with glyceryl trinitrate in hypertensive stroke trial (RIGHT), reported that paramedics’ motivation to participate in stroke research was a facilitator to undertaking more pre-hospital stroke research. These findings suggest that paramedics want to base decisions on existing research and be part of producing research to build the evidence base.

### Strengths and limitations

The systematic approach of the framework method (Ritchie & Lewis, 2003) provides a sufficient, clear process and the analysis process was able to be transparently communicated in the methods section. Using more than one researcher to analyse the data and code the transcripts provided credibility to the findings and added breadth to the understanding of the issues (Carter et al., 2014).

While the interviewed paramedics had diverse backgrounds in terms of age, training and experience, they were all of the same ethnicity and pre-dominantly male. It is unclear whether a more diverse group would have had shared or divergent views on the use of PHECG for stroke patients, especially considering the influence of patient sex on the paramedics’ decision-making. This highlights the need for future studies to include a more diverse sample to provide a more comprehensive understanding of the ethical concerns surrounding the use of PHECG for stroke patients.

## Conclusion

In conclusion, this study presents a novel exploration of the perspectives and practices of paramedics involved in the care of acute stroke patients and the use of PHECGs. While mixed views were expressed among participants regarding the importance, usefulness and relevance of PHECGs for pre-hospital management of stroke patients, all participants agreed that it should not lead to additional delays in reaching the hospital.

This study is unique in its use of CCT to investigate paramedic decision-making processes. The findings demonstrate the potential of CCT to understand the decision-making rationale underlying paramedic actions. The authors believe that further application of CCT could make contributions to research on paramedic decision-making.

## Acknowledgements

We would like to thank all the participants for their involvement in this study.

## Author contributions

SM designed and conceptualised the study, analysed the data and drafted the manuscript for intellectual content. DC designed and conceptualised the study, interpreted the data and revised the manuscript for intellectual content. JH was a lay representative, who designed the study and revised the manuscript for intellectual content. TQ designed and conceptualised the study, interpreted the data and revised the manuscript for intellectual content. All authors read and approved the final manuscript. SM acts as the guarantor for this article.

## Conflict of interest

TQ reports grants from the National Institute for Health Research and British Heart Foundation. All other authors declare no conflict of interest.

## Ethics

Favourable ethical approval was gained from NHS Research Ethics Committee Berkshire B, and Health Research Authority approval was granted (REC ref 16/SC/0528). Written informed consent was gained from all participants who were interviewed.

## Funding

This work was supported by a School of Health Sciences PhD bursary – University of Surrey, and South East Coast Ambulance Service NHS Foundation Trust.
